# Dissecting Migraine: The Future of Anatomical, Functional, and Liquid Biomarkers

**DOI:** 10.3390/jcm11195538

**Published:** 2022-09-21

**Authors:** Ciro De Luca, Filippo Baldacci

**Affiliations:** 1 Laboratory of Morphology of Neuronal Networks and Systems Biology, Department of Mental and Physical Health and Preventive Medicine, University of Campania “Luigi Vanvitelli”, 80138 Napoli, Italy; 2 Neurology Unit, Department of Clinical and Experimental Medicine, University of Pisa, 56126 Pisa, Italy

Migraine is a common yet enigmatic disease that, despite its high prevalence and familial presentation, lacks exhaustive genetic or environmental causative factors. It is the most prevalent neurological disease and the primary determiner of years lived with disability among the young population worldwide, and it can be diagnosed and followed up exclusively through clinical criteria, collected in the third edition of the International Classification of Headache Disorders (ICHD-3) [1,2]. Indeed, migraine, understood as a spectrum of clinical syndromes (migraine with or without aura, chronic migraine, etc.), has no reliable biomarker for establishing the correct diagnosis, disease progression, or therapeutical response. Moreover, headache is also the principal manifestation of very common primary headaches (e.g., tension-type headache—TTH) and secondary neurological diseases (e.g., subarachnoid hemorrhage), which span from harmless to life-threatening, making the development of specific biomarkers paramount.

Patients can also fit multiple diagnostic criteria, and in some cases, diagnoses often overlap, as is the case for TTH and migraine, during the follow-up of a single patient. Conversely, entities with the same clinical diagnosis, such as treatable low-frequency episodic migraine and drug-resistant high-frequency episodic migraine, represent completely different therapeutical challenges. The development of predictive biomarkers for migraine transformation can aid in preventing it by intercepting susceptible subjects.

The anatomical basis of migraine conveys peripheral harmless stimuli from the trigeminal nerve, coupled with meningeal vessels (trigeminovascular pathway) to be relayed into the thalamus and interpreted consciously as painful [3]. However, in migraine, the genesis of the cyclic pain, interictal symptoms, and associated comorbidities probably depend on morphofunctional modifications of the brainstem (the spinal trigeminal nucleus, periaqueductal gray, and ventral tegmental area) and diencephalic regions (hypothalamus) that contribute to the alteration of cortical excitability.

Aside from the conflicting results of morphology, functionality, imaging, and molecular tracing studies have given a general understanding of the networks that are differently impaired in migraine pathophysiology. However, these data have not been translated in terms of consistent advances in diagnosis or prognosis so far [4].

The biomolecular challenge was paved with the search for the “substance of pain”, which is very controversial due to the lack of a good animal model for the disease. The key molecule substance P involved in neurogenic inflammatory pain and the antidromic signaling of sensory axons represent an unreliable target in the clinical translation of the results. The revolution of migraine biomarkers was brought about by the calcitonin gene-related peptide (CGRP) and the associated receptor and co-receptor, which are currently the most prevalent topic in headache research [5,6]. CGRP, collected from the jugular vein after the headache attack, is the most reliable biomarker to be found but is useless in the clinical setup and in large cohorts of patients due to its invasiveness and the need for a strict timing of sampling.

The emergent role of CGRP has been paralleled by changes in the experimental settings, whereas the induction of migraine-like attacks with nitric oxide has been substituted by the more specific molecule. However, CGRP can reproduce a headache in only 77% of patients. The adenosine 5′-triphosphate-sensitive K^+^ (K_ATP_) channel seems to be more powerful. Levcromakalim infusion opening the K_ATP_ channel provokes migraine attacks associated with the vasodilation of extracerebral arteries in all migraine patients [7]. This discovery has opened a very important field of investigation and has probably found the final common pathway for the development of migraine attack, which can be downstream of CGRP or activated independently through other receptors. The common activation of metabolic pathways towards dysfunctional cortical hyperactivity and metabolic failure was conducive to the exploration of the redox state as a putative biomarker [5,8]. However, even if the increase in plasma biomarkers of oxidative stress is related to migraine disability and chronicity, more data are needed to consider it predictive for migraine diagnosis or response to treatments.

The research on plasma and other biomarkers has not had promising results, particularly considering the genotyping of migraine patients [4]. However, non-invasive and less canonical approaches could give an answer where more powerful instruments have failed.

Values of intracranial blood flow, measured through transcranial Doppler, showed encouraging results, considering asymmetric velocities of arterial circulation in paired vessels (right-lower compared to left) and lower velocities measured in the basilar artery expressed by migraine patients responding to anti-CGRP treatment. Moreover, the treatment itself with Erenumab normalized the measured values. This study should be replicated before drawing definite results but could offer a non-invasive, replicable, and consistent tool to predict drug response, further validating the trigeminovascular hypothesis [6].

The sensory pathways other than the fifth cranial nerve have also been explored, assessing the peripheral auditory system in migraineurs using distortion product otoacoustic emissions (DPOAEs) [9]. DPOAE alterations and suppression were related to disease risk, diagnosis, duration, and characteristics such as concomitant dopaminergic symptoms, allodynia, and usage of painkillers. Hence, subclinical cochlear impairment detected by DPOAEs could provide an easy and cheap instrument for the confirmation of clinical diagnosis and be useful to monitor the course of the disease.

Furthermore, the visual system, anatomic, and functional pathway from the retina to the primary visual cortex was also investigated as a biomarker using visual evoked potentials (VEP). The saturation index was used to record the response of the VEP’s amplitude to the contrast gain. Patients with episodic migraine showed non-linear or monotone growth of the saturation, defined as supersaturation. Supersaturation was consistently expressed in migraineurs, and a strong inverse correlation was found between the saturation index and the number of days separating the registration of VEP from the next migraine attack. Moreover, allodynia correlated with the saturation of the index that was not influenced by the treatment with topiramate. This pilot study described an electrophysiological biomarker of the retino-thalamo-cortical excitability in the migraine cycle associated with the ictal phase of the migraine. Moreover, the excitability of somatosensory and visual cortices seems to be consistently related [10].

Non-conventional and complementary approaches to the treatment of migraine have also been investigated. These treatments paralleled with the recent introduction of targeted drugs and other evidence-based prophylactic therapies could aid clinicians in managing either drug-resistant or low-frequency episodic migraineurs. In other words, a non-pharmacological approach can be used where other therapies have failed or where the risk-to-benefit ratio is not favorable.

The ketogenic diet has gained attention as a preventive treatment for migraine, sustained by pre-clinical and clinical data, although it still controversial. In the short term, it has been reported as being associated with a reduction in headache frequency and painkillers, coupled with weight loss and a reduction in fat mass. The putative mechanism has not been elucidated; however, if a ketogenic diet is indicated in the patient for other health reasons, it could add more data about the effects of dietary conduits on headaches [11].

Musculoskeletal and soft-tissue manipulations have also been studied for their feedback on pain perception and theories of peripherally gated afferent signaling. The relationship between neck stiffness and headache, particularly migraine, has been largely recognized clinically. Moreover, exteroceptive non-nociceptive signaling could potentially interfere with the trigeminovascular dysfunction of migraine pathophysiology. Indeed, the manual therapy protocol of soft tissue and articulations reduced pain, disability, and the associated affective symptoms (depression or anxiety) in migraine patients [12].

Most migraine-related studies, however, target a homogenous young population. The presence of these experimental cohorts makes it difficult to deal with other categories of patients also experiencing similar clinical syndromes. It could be important to focus on the elderly, understanding the prevalence of the disease, clinical differences, response to treatment, and social factors, or on how the same disease could vary considering hormonal changes in young and post-menopause women [13,14]. In particular, the presence of quantifiable proteins from accessible body fluids such as urine could lead to a specific understanding of the atypical presentation of primary headache in the elderly.

Taken together, these studies collected novel, out-of-the-box projects on migraine diagnosis, prognosis, treatment, and prevalence. Where the canonical approaches to big data genotyping and targeted signaling failed to reach satisfactory results, novel ideas of electrophysiological, vascular, redox, and urinary biomarkers can furnish a more complex and satisfactory picture of the most disabling primary headache.

## Data Availability

Not applicable.
